# Seasonal characteristics of phosphorus sorption by sediments from plain lakes with different trophic statuses

**DOI:** 10.1098/rsos.172237

**Published:** 2018-08-15

**Authors:** Wei Huang, Xing Chen, Kun Wang, Xia Jiang

**Affiliations:** 1 National Engineering Laboratory for Lake Pollution Control and Ecological Restoration, Chinese Research Academy of Environmental Sciences, Beijing, People's Republic of China; 2 State Key Laboratory of Environmental Criteria and Risk Assessment, Chinese Research Academy of Environmental Sciences, Beijing, People's Republic of China

**Keywords:** trophic status, sorption, phosphorus, sediment, lake

## Abstract

Phosphorus (P) sorption in sediments plays a significant role in trophic status of a lake. This study investigated the characteristics of P sorption in sediments from three lakes with different trophic statuses (moderately eutrophic, lightly eutrophic and moderately trophic) through kinetic, batch equilibrium and thermodynamic experiments. Results show that pseudo-second-order kinetics best describe P sorption in sediments from the three lakes. Fitting by modified Langmuir and Freundlich isotherms indicates that the moderately trophic lake sediment has higher sorption capacity (maximum of 0.848 mg g^−1^ at 35°C) than the sediments of the other two lakes at different temperatures (5, 15, 25 and 35°C). Thermodynamic results indicate that the processes of P sorption of the three sediments are spontaneous, entropy-driven and endothermic reactions. The risk of P release in sediments was analysed according to the calculated results of isotherms combined with the change in P fraction. Sediments from the moderately eutrophic lake act as a source in summer. The lightly eutrophic and moderately trophic lakes act as sources in spring and winter, and a pool in summer and autumn, respectively. Furthermore, the amounts of reductant-soluble P, calcium-bound P and iron-bound P are significantly related to the sorption capacity of sediments from the three lakes (*p* < 0.05). The different sediments have different P release risk, and P fraction in sediment is one of the significant factors of P sorption.

## 1. Introduction

Eutrophication is one of the most serious environmental problems in lakes, especially for shallow lakes. Phosphorus (P) is a key nutrient in eutrophication, and excessive P in water plays a crucial role in eutrophication of lakes, which can result in the chaotic growth of undesirable algae or the bloom of aquatic plants [1–4]. In lake sediments, P also exists in different forms (e.g. calcium, iron, or aluminium complex salts and organic species), and P in sediment may have an influence on the P concentration in a lake. The trophic status, one of the most significant indices of lake eutrophication, is commonly defined as the structural and functional quality of water bodies. This parameter is assessed using different indices, such as biological communities, combined with physical, chemical, morphological and hydrological characteristics in various aquatic ecosystems [5,6]. The composition, sorption and release of P in sediment can directly influence P concentration in lakes and may cause a shift in trophic status [7].

In the water column, P concentration is the main parameter used to classify bodies of water according to trophic status (oligotrophic, mesotrophic or eutrophic). Lake sediment substantially accumulates nutrients, and can be seen as a general porous medium storing a variety of nutrients. Trophic status modulates sediment functions to supply nutrients, and in turn, is greatly affected by the sediment in shallow lakes [8]. As one of the main factors that influence the P concentration in lakes, P sorption of the sediment has different characteristics because of the different conditions in the lake [9,10], which can cause the change of the trophic status. The characteristics of P sorption, such as the ability of sediment to act as a pool or a source of P, depend on the physicochemical (particle size, porosity, nutrient contents, etc.) and environmental conditions (temperature, ionic strength, pH, P concentration in water body, etc.), which are regarded as the main factors affecting P release flux through the sediment–water interface [11,12]. In the plain geomorphological region, shallow freshwater lakes with different trophic status exist commonly because of various factors. As an internal source, P sorption in sediments may influence the P concentration in water, and the effect of P sorption on the trophic status of lakes should not be ignored. The sorption characteristics of P at the sediment–water interface are therefore significant to study [1,13].

In the Eastern Plain of China, a large number of shallow, freshwater lakes with different trophic status exist, and the sediments in these lakes have a great influence on their trophic status. The regularity of P sorption from sediments in these lakes is closely related to their trophic status [14,15]. Gonghu Bay is an algae-dominated region located in the northeast part of Taihu Lake and covers an area of 147 km^2^, with a mean depth of 2.0 m. Gonghu Bay has been a moderately eutrophic lake with a trophic state index (TSI) of 64.8 because of the great increase in frequency of algal bloom [16–18]. Another large freshwater lake, Dongting Lake, is divided into three sections, namely, eastern, southern and western. The East Dongting Lake has been seriously contaminated, and extensive algal blooms or occurrences of aquatic organism death were observed in recent years with TSI of 52.6 (lightly eutrophic region). Huangda Lake, with an area of 299 km^2^, has an average depth of 3.64 m. Huangda Lake is a clean lake with TSI value of 45.4 in recent years. In addition, the Eastern Plain of China has four distinctive seasons, and these three lakes have an obvious temperature variation in each season.

Therefore, the objective of this study was to analyse the influence of the season on P sorption characteristics in sediment from regions with different trophic status, and study the mechanism of P sorption in sediment. In the present study, the sediment samples from three regions with different trophic status (moderately eutrophic, lightly eutrophic and moderately trophic) were chosen, and the P sorption characteristics (kinetics, isotherms and thermodynamic) of the sediments in three lakes were compared with each other. The changes of P fractions in sediments from three lakes following sorption and the pool or source of P were analysed to evaluate the risk of P release.

## 2. Material and methods

### 2.1. Sediment sampling and analysis of physicochemical parameters

Sediment samples for sorption experiments were collected from Gonghu Bay of Taihu Lake (T, N30°27 ′24″, E116°18′13″), East Dongting Lake (DT, N33°57′29″, E118°15′20″) and Huangda Lake (HD, N31°22′50″, E120°10′15″) in four seasons (April, August, October and December) in 2016, as well as the overlying water in these three lakes. All the sediment or water samples were from the polluted areas of the three lakes. Five composite surface (0–10 cm) sediment samples and three parallel samples of the overlying water were collected at the sampling sites using a grab sediment sampler and water sampler, respectively. The sediment samples were immediately moved to the laboratory, where they were freeze-dried, ground and passed through a 100-mesh (0.15 mm) sieve to obtain uniform size [19]. The dry samples obtained were used in all of the experiments of this study. The experimental temperatures of 5, 15, 25 and 35°C were chosen corresponding the seasons of winter, spring, autumn and summer, respectively.

The total phosphorus and nitrogen in sediment were measured by using the standard method, and ammonium by colorimetry [20,21]. The phosphorus concentration in the extracted solution or water body was determined with the molybdenum blue method after the solution was filtered (0.45 µm filtration membrane) [22]. The pH of the sediment was measured in a 1 : 2.5 (w/v) mixture of sediment with deionized water [23]. Organic matter (OM) content was calculated according to the loss on ignition to constant mass (4 h) at 550°C [1]. The Fe, Ca and Al oxide contents were determined using an X-ray fluorescence analyser (S4 EXPLORER, Germany). The physicochemical parameters are shown in table 1.

**Table 1.** Physicochemical properties (mean values ± standard deviation) of the sediments from three lakes. Note: T, DT and HD stand for the sediments from Gonghu Bay, East Dongting Lake and Huangda Lake, respectively. The mean values were calculated from the data of the sediments from four seasons.

property (unit)	T	DT	HD
TN (mg kg^−1^)	1708.1 ± 83.2	912.6 ± 78.5	1969.5 ± 118.2
TP (mg kg^−1^)	793.1 ± 67.2	823.6 ± 66.9	571.9 ± 72.4
OM (%)	5.67 ± 0.45	9.32 ± 0.67	9.51 ± 0.72
pH [H_2_O]	7.85 ± 0.56	7.15 ± 0.63	6.21 ± 0.66
Fe oxide (%)	7.38 ± 0.82	8.23 ± 0.72	11.34 ± 0.71
Ca oxide (%)	13.21 ± 0.92	26.33 ± 1.23	16.26 ± 1.82
Al oxide (%)	11.23 ± 1.56	11.54 ± 1.03	12.83 ± 1.65

### 2.2. Kinetic sorption experiments

Batch experiments were carried out to obtain the kinetics of P sorption. The stock solution, which was obtained by dissolving the salt KH_2_PO_4_ in deionized water, was prepared within 24 h and was stored in the dark at 4°C before use. Sediment samples (0.2 g) were added into 100 ml of 20 mg l^−1^ P solution. Samples collected in four seasons were shaken in a temperature-controlled shaker under various constant temperatures of at 5, 15, 25 and 35°C at a speed of 220 rpm in batches. The equilibration time used to evaluate the sorption kinetics is 1, 2, 4, 8, 10, 20, 40, 60, 120, 240, 360 and 480 min. The suspensions were taken from each flask, centrifuged, filtered (0.45 µm) and the P concentration was analysed by the molybdenum blue method [22]. The experiments were conducted in triplicate, and the sediment sample (2 g) to solution (100 ml) ratio was assumed constant throughout the incubation period.

The P sorption capacity of the sediment samples at each time, *Q_t_* (mg g^−1^), was calculated by using a mass balance relationship as follows:

2.1 $$Q_{t}=(C_{0}-C_{t})\frac{V}{W},$$

where *C*_0_ (mg l^−1^) is the initial liquid-phase P concentration, *C_t_* (mg l^−1^) the blank corrected concentration of P at time *t*, *V* (l) the volume of the solution and *W* (*g*) the mass of dried sediment samples.

Sorption kinetics is described by pseudo first- and second-order models, and the models are expressed as follows:

2.2 $$Q_{t}=Q_{e}(1-{\text{e}}^{-K_{1}t})$$

and

2.3 $$\frac{t}{Q_{t}}=\frac{1}{K_{2}Q_{e}^{2}}+\frac{t}{Q_{e}},$$

where *Q*_e_ and *Q_t_* were the amounts (mg g^−1^) of P adsorbed at equilibrium time and at time *t* (min), respectively; *K*_1_ was the first-order kinetics constant (min^−1^); *K*_2_ was the second-order kinetics constant (g mg^−1^ min^−1^) [24,25].

### 2.3. Sorption isotherm experiments

The sorption isotherms experiments were conducted at 5, 15, 25 and 35°C. Samples (0.2 g) were added to a series of beaker flasks containing 10 ml P solutions with various P concentrations (0, 1, 2, 5, 10, 20 and 50 mg l^−1^). The beaker flasks were shaken in a temperature-controlled shaker at a speed of 220 r.p.m. under the various constant temperatures. After 480 min, the solution was filtered (0.45 µm filtration membrane) for P analysis.

Modified Langmuir and Freundlich models were used to describe the sorption isotherms, and the isotherm parameters are expressed as follows [10]:

2.4 $$Q_{e}=\frac{Q_{m}KC_{e}}{1+KC_{e}}\;-\;\frac{Q_{m}KC_{e}^{0}}{1+KC_{e}^{0}}-\;Q_{e}^{0},$$

2.5 $$\text{NAP = }\frac{Q_{m}KC_{e}^{0}}{1+KC_{e}^{0}}+Q_{e}^{0}$$

2.6 $$\;\text{and}\;\text{EP}{\text{C}}_{0}=\frac{\text{NAP}}{K(Q_{m}-\;\mathrm{NAP})},$$

where *Q*_e_ and *Q*_m_ are the adsorbed amounts of P in the sediments at equilibrium and the maximum P uptake amount (mg g^−1^), respectively. *C*_e_ is the P concentration in the aqueous phase at equilibrium (mg l^−1^), and *K* is the affinity parameter (l mg^−1^). *C*_e_^0^ and *Q*_e_^0^ are the equilibrium concentration (mg l^−1^) and uptake amount (mg g^−1^), respectively, and *C*_add_ (initial concentration of newly added P in the solution of sorption trials) is equal to 0 mg l^−1^. The zero equilibrium P concentration (EPC_0_) is the concentration (mg l^−1^) in which no net sorption or desorption of P occurs, and the original sediment and water P concentrations are in dynamic equilibrium.

The modified Freundlich model can be derived through the same method, and the equations of the isotherm parameters are expressed as follows [26]:

2.7 $$Q_{e}=K_{f}C_{e}^{m}\;-K_{f}{\left(C_{e}^{0}\right)}^{m}\;-\;Q_{e}^{0},$$

2.8 $$\text{NAP = }K_{f}{\left(C_{e}^{0}\right)}^{m}+Q_{e}^{0},$$

2.9 $$\text{EP}{\text{C}}_{0}=\sqrt[{m}]{\frac{\text{NAP}}{K_{f}}}$$

2.10 $$\;\text{and}\;K_{p}=\frac{\text{NAP}}{\text{EP}{\text{C}}_{0}},$$

where *K*_f_ is the sorption coefficient (l g^−1^) and *m* a constant used to measure sorption intensity or surface heterogeneity. *K*_p_ (l g^−1^) is the affinity parameter of the modified Langmuir model.

### 2.4. Thermodynamic parameters

Samples (0.2 g) were added into 100 ml P solutions with various initial concentrations (0, 1, 2, 5, 10, 20 and 50 mg l^−1^) at four different temperatures: 5, 15, 25 and 35°C. Batch samples were shaken in a temperature-controlled shaker for 480 min. The thermodynamic parameters of P sorption, such as enthalpy (Δ*H*), Gibbs energy (Δ*G*) and entropy (Δ*S*), were estimated by fitting linear equations to the thermodynamic data obtained under different concentrations.

The thermodynamic parameters were analysed with the following equations [27,28]:

2.11 $$K_{D}=\frac{C_{0}-C_{e}}{C_{e}}\times \frac{V}{m},$$

2.12 $$\Delta G^{0}=-RT\text{ln( }K_{D})$$

2.13 $$\;\text{and}\;\text{ln}(K_{D})=\frac{\Delta H^{0}}{RT}+\frac{\Delta S^{0}}{R}.$$

### 2.5. Analysis of P fraction

The content of the P fraction in sediment after sorption was determined in order to analyse the distribution of adsorbed P onto the sediments. The following sequential extractions were performed after equilibrating sediments with an initial 20 mg l^−1^ P solution at the temperature of 25°C. The soluble and loosely bound P (S/L-P) was removed using ammonium chloride (1 mol l^−1^ NH_4_Cl). Aluminium-bound P (Al–P) was determined by the separation from iron-bound P (Fe–P) using NH_4_F (0.5 mol l^−1^), and Fe–P was extracted using NaOH (0.1 mol l^−1^). Calcium-bound P (Ca–P) was extracted by H_2_SO_4_ solution (0.25 mol l^−1^). Reductant-soluble P (RS-P) in sediments was removed using CDB (0.3 mol l^−1^ sodium citrate (Na_3_C_6_H_5_O_7_·2H_2_O)/25 g l^−1^ sodium dithionite (NaS_2_O_4_)/1 mol l^−1^ sodium bicarbonate) extraction [29,30].

### 2.6. Statistical analysis

The relationship between P fraction and P sorption capacity of the sediments was investigated using analysis of variance with SPSS v.21.0.

## 3. Results

### 3.1. Phosphorus sorption kinetics

**Figure 1.**
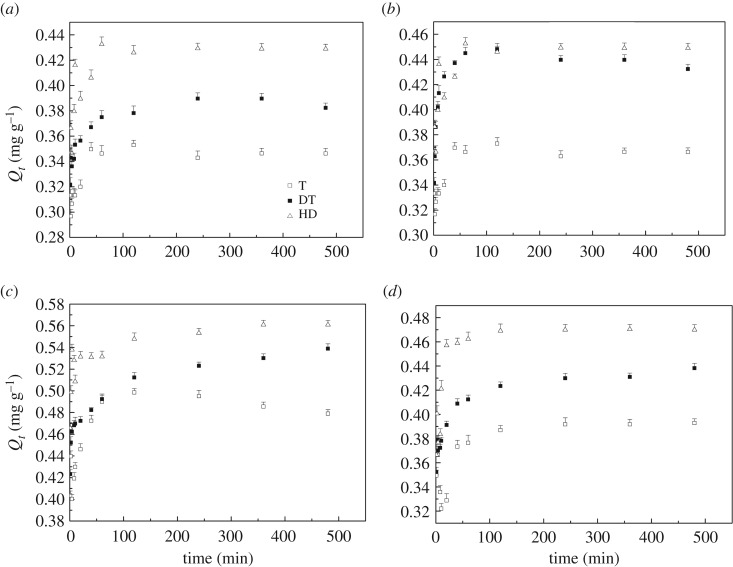
Sorption kinetics of P on three sediments at (*a*) 5°C (winter), (*b*) 15°C (spring), (*c*) 25°C (autumn) and (*d*) 35°C (summer).

The P sorption kinetics of three sediments at different temperatures, and the experiment results corresponding to the P sorption fitting the kinetic equations (equations (2.2) and (2.3)) are shown in figure 1 and table 2, respectively. The kinetic behaviour of P sorption in the three sediments was examined in a 480 min contact time at various temperatures (5, 15, 25 and 35°C). The sorption velocity of the three sediments was fast at the beginning (60 min), but turned slow immediately after the initial sorption. The moderately trophic lake, Huangda Lake, had the highest sorption capacity, whereas Gonghu Bay of Taihu Lake had the lowest. The sorption capacities increased as the temperature increased from 5 to 25°C, and then decreased at 35°C. In Huangda Lake, the relatively high sorption capacities of the sediments vary from 0.431 to 0.562 mg g^−1^ at different temperatures, and the sediments from Gonghu Bay of Taihu Lake and East Dongting Lake had the largest sorption capacity of 0.493 and 0.531 mg g^−1^ at 2°C, respectively.

**Table 2.** Parameters for pseudo-first-order and pseudo-second-order kinetics of P sorption at different temperatures. Note: T, DT and HD stand for the sediments from Gonghu Bay, East Dongting Lake and Huangda Lake, respectively.

temperature	sample	pseudo-first-order			pseudo-second-order		
		*Q*_e_	*K*_1_	*R*^2^	*Q*_e_	*K*_2_	*R*^2^
5°C (winter)	T	0.278 ± 0.007^a^	2.455 ± 0.178	0.9376	0.289 ± 0.007	8.148 ± 1.127	0.9834
	DT	0.348 ± 0.012	1.765 ± 0.204	0.9436	0.325 ± 0.009	6.328 ± 0.967	0.9729
	HD	0.376 ± 0.005	1.424 ± 0.311	0.9568	0.410 ± 0.011	5.987 ± 1.076	0.9673
15°C (spring)	T	0.351 ± 0.006	2.785 ± 0.238	0.9621	0.357 ± 0.005	20.018 ± 2.057	0.9754
	DT	0.373 ± 0.010	2.305 ± 0.198	0.9322	0.391 ± 0.009	12.018 ± 1.257	0.9623
	HD	0.427 ± 0.008	1.799 ± 0.391	0.9493	0.437 ± 0.007	8.889 ± 1.413	0.9738
25°C (autumn)	T	0.461 ± 0.010	2.021 ± 0.237	0.9428	0.471 ± 0.009	9.721 ± 3.618	0.9599
	DT	0.498 ± 0.013	2.085 ± 0.218	0.9765	0.447 ± 0.015	8.238 ± 1.347	0.9858
	HD	0.539 ± 0.006	1.886 ± 0.237	0.9846	0.547 ± 0.005	10.183 ± 2.139	0.9916
35°C (summer)	T	0.367 ± 0.008	3.059 ± 0.229	0.9422	0.370 ± 0.008	39.351 ± 3.596	0.9453
	DT	0.410 ± 0.009	2.285 ± 0.247	0.9562	0.389 ± 0.011	18.123 ± 2.873	0.9741
	HD	0.442 ± 0.011	1.618 ± 0.184	0.9283	0.455 ± 0.009	6.751 ± 1.926	0.9617

^a^Values are mean ± standard deviation, *n* = 3.

Table 2 describes the rate constants and parameters of the pseudo-first- and pseudo-second-order models derived from the nonlinear regression. Results showed that a pseudo-second-order model (*R*^2^ = 0.9453–0.9916) can better describe the P sorption kinetics than pseudo-first-order model (*R*^2^ = 0.9283–0.9846) at four different temperatures. The sediments had the highest equilibrium sorption capacity (*Q*_e_) at 25°C, and the sediments from Huangda Lake had the highest values of 0.547 mg g^−1^. In addition, the moderately eutrophic lake, Gonghu Bay of Taihu Lake, had a higher *Q*_e_ than the other two lakes, and the lowest *Q*_e_ was 0.289 mg g^−1^ at 5°C. As a whole, the *Q*_e_ values were such that the moderately eutrophic lake < lightly eutrophic lake < moderately trophic lake at different temperatures, and the sediments from three lakes had the highest *Q*_e_ at 25°C.

### 3.2. Sorption isotherms

**Figure 2.**
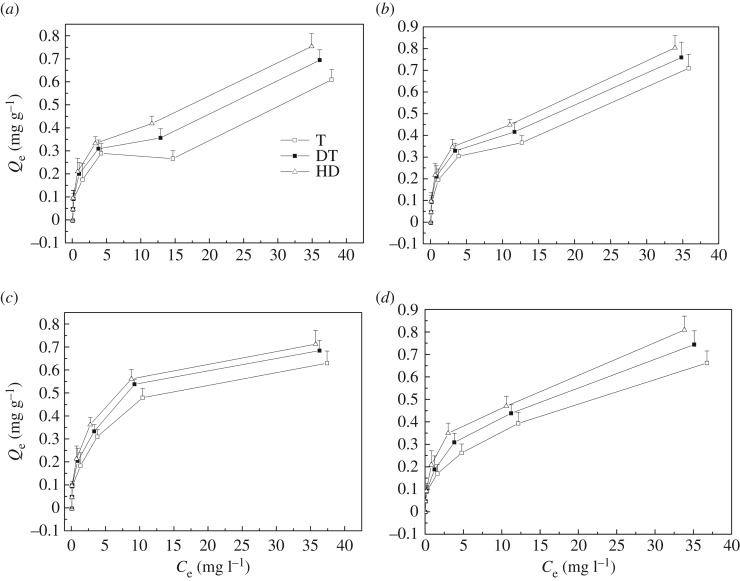
Sorption isotherms of P on three sediments at different temperatures: (*a*) for 5°C (winter), (*b*) for 15°C (spring), (*c*) for 25°C (autumn), and (*d*) for 35°C (summer).

In this study, the sediment isotherms were described by modified Langmuir and Freundlich models (equations (2.4) and (2.7)). The isotherms and the fitting data are exhibited in figure 2 and table 3. As evidenced by the correlation coefficient *R*^2^, the P sorption isotherms of the three sediments can be described by using a modified Langmuir model (*R*^2^ = 0.9442–0.9974) rather than using the modified Freundlich model (*R*^2^ = 0.8541–0.9553). The Langmuir sorption isotherm provided a good estimate of the theoretical sorption maxima (*Q*_m_) to reflect the sorption capacity of the sediments from lakes with different trophic status. The *Q*_m_ of the sediments from the moderately eutrophic, lightly eutrophic and moderately trophic lakes ranged within 0.547–0.817, 0.632–0.819 and 0.729–0.848 mg g^−1^ at different temperatures, respectively. The *Q*_m_ of the sediment from Huangda Lake had higher value than other two sediments at different temperatures, and the value reached 0.848 mg g^−1^ at 35°C. The affinity parameter, *K*, calculated from the modified Langmuir model had a similar trend as the *Q*_m_. *K* values rose as the temperature increased, and the moderately trophic lake, Huangda Lake, had the highest *K* values at different temperatures.

**Table 3.** Modified Langmuir and Freundlich model parameters for P sorption. Note: T, DT and HD stand for the sediments from Gonghu Bay, East Dongting Lake and Huangda Lake, respectively.

temperature	sample	modified Langmuir: $Q_{e}=\frac{Q_{m}KC_{e}}{1+KC_{e}}-\frac{Q_{m}KC_{e}^{0}}{1+KC_{e}^{0}}-Q_{e}^{0}$						modified Freundlich: $Q_{e}=K_{f}C_{e}^{n}-K_{f}{\left(C_{e}^{0}\right)}^{n}-Q_{e}^{0}$					
		fitting results			calculated results			fitting results			calculated results		
		*K* (l mg^−^1)	*Q*_m_ (mg g^−1^)	*R*^2^	NAP (mg g^−1^)	EPC_0_ (mg l^−1^)	*K*_p_ (l g^−1^)	*K*_f_ (l g^−1^)	*m*	*R*^2^	NAP (mg g^−1^)	EPC_0_(mg l^−1^)	*K*_p_ (l g^−1^)
5°C (winter)	T	0.123	0.573	0.9442	0.010	0.139	0.069	0.128	0.089	0.8641	0.106	0.124	0.855
	DT	0.157	0.632	0.9444	0.010	0.107	0.098	0.156	0.104	0.9023	0.122	0.094	1.303
	HD	0.201	0.729	0.9677	0.008	0.055	0.145	0.211	0.123	0.8813	0.144	0.046	3.154
15°C (spring)	T	0.145	0.779	0.9443	0.011	0.103	0.111	0.282	0.292	0.8646	0.134	0.077	1.727
	DT	0.186	0.838	0.9623	0.012	0.081	0.154	0.312	0.285	0.8923	0.143	0.065	2.196
	HD	0.217	0.833	0.9671	0.007	0.039	0.179	0.356	0.266	0.8813	0.143	0.032	4.447
25°C (autumn)	T	0.252	0.709	0.9599	0.018	0.104	0.174	0.594	0.159	0.9553	0.402	0.085	4.703
	DT	0.323	0.810	0.9672	0.022	0.087	0.254	1.324	0.232	0.9123	0.723	0.074	9.792
	HD	0.438	0.769	0.9572	0.025	0.041	0.326	1.405	0.081	0.9537	1.131	0.068	16.524
35°C (summer)	T	0.100	0.817	0.9894	0.004	0.053	0.081	0.186	0.377	0.9458	0.053	0.036	1.485
	DT	1.239	0.839	0.9886	0.025	0.025	1.009	0.156	0.412	0.9223	0.034	0.025	1.351
	HD	2.015	0.848	0.9974	0.021	0.013	1.666	0.277	0.32	0.9243	0.068	0.013	5.420

In order to evaluate the P release risk, the EPC_0_ and NAP values were calculated using the modified Langmuir model. The same sediment had different EPC_0_ and NAP values (table 3) at different temperatures. The EPC_0_ of different sediments decreased as the temperature increased; the sediments from Gonghu Bay of Taihu Lake had the highest EPC_0_ of 0.139 mg l^−1^ at 5°C. The NAP of the sediments had low values at 5 and 15°C, whereas the sediments had high values at 25 and 35°C. At 5, 15 and 25°C, the sediments from the three lakes had similar NAP values, and Huangda Lake had the lowest NAP of 0.04 mg g^−1^ at 35°C.

### 3.3. Thermodynamic parameters

The thermodynamic parameters were calculated by using equations (2.11)–(2.13), and the result is shown in table 4. The Δ*S*^0^ values of the three sediments had few changes at different temperatures and initial P concentrations and were all lower than 0.10 kJ mol^−1^. The Δ*G*^0^ values of these three sediments are negative and decreased as the temperature increased. As the initial P concentration increased, the Δ*G*^0^ values also increased. The Δ*G*^0^ ranged from −6.42 to −16.29 kJ mol^−1^ in Gonghu Bay of Taihu Lake and from −6.83 to −16.92 kJ mol^−1^ in East Dongting Lake at different initial P concentrations. The sediment from Huangda Lake had Δ*G*^0^ values which ranged from −7.10 to −17.36 kJ mol^−1^. The Δ*H*^0^ values were negative at the lowest and highest initial P concentrations, and were positive at other initial P concentrations.

**Table 4.** Thermodynamic parameters for P sorption at different temperatures and initial P concentration. Note: *C*_0_ (mg l^−1^) is the initial liquid-phase P concentration; Δ*H*^0^ (kJ mol^−1^) is apparent heat of sorption; Δ*S*^0^ (kJ mol^−1^ K^−1^) is entropy change; Δ*G*^0^ (kJ mol^−1^) is change of Gibbs free energy. T, DT and HD stand for the sediments from Gonghu Bay, East Dongting Lake and Huangda Lake, respectively.

*C*_0_	T						DT						HD					
	Δ*S*^0^	Δ*H*^0^	Δ*G*^0^				Δ*S*^0^	Δ*H*^0^	Δ*G*^0^				Δ*S*^0^	Δ*H*^0^	Δ*G*^0^			
			5°C	15°C	25°C	35°C			5°C	15°C	25°C	35°C			5°C	15°C	25°C	35°C
1	0.09	−10.23	−13.63	−14.61	−15.23	−16.29	0.08	−9.40	−14.26	−14.89	−15.48	−16.92	0.10	−12.52	−14.31	−15.17	−15.74	−17.36
2	0.05	0.52	−14.24	−15.20	−16.19	−15.52	0.05	2.13	−14.67	−16.15	−16.91	−15.87	0.03	6.38	−15.37	−16.73	−17.52	−16.13
5	0.03	3.59	−11.00	−12.40	−12.20	−11.94	0.02	6.76	−12.25	−13.34	−13.37	−12.87	0.04	1.96	−12.90	−13.91	−14.03	−14.17
10	0.02	4.16	−9.77	−10.42	−10.90	−10.25	0.04	0.39	−10.15	−10.93	−11.40	−11.25	0.06	4.58	−10.62	−11.33	−12.09	−12.17
20	0.08	16.04	−6.70	−8.05	−9.48	−8.90	0.07	11.11	−7.67	−8.56	−10.07	−9.38	0.06	7.84	−8.29	−8.88	−10.30	−9.71
50	0.03	−1.28	−6.42	−7.15	−6.99	−7.40	0.03	−1.05	−6.83	−7.38	−7.27	−7.82	0.03	−0.89	−7.10	−7.58	−7.41	−8.13

### 3.4. Changes of P fractions in sediment following sorption

The P fraction transformation in control sediments and in these sediments after P sorption was different in sediments from lakes with different trophic status. The sediments in autumn were chosen to analyse the P fraction transformation because of the high sorption capacity of the sediment in autumn. Table 5 shows the distribution of P fraction before and after sorption in control sediments. Results showed that in sediments from three lakes with different trophic status, a significant amount of S/L-P and Al–P were observed in sediment after P sorption compared with the control sediments. The percentage of adsorbed S/L-P reached 49.5, 31.9 and 44.4% in sediments from moderately eutrophic, lightly eutrophic and moderately trophic lakes, respectively, and S/L-P might be the main P fraction in sorption. *Q*_m_ also had a significant relationship with S/L-P in these three sediments (*p* < 0.05). In addition, the transformation of P fraction in sediments from lakes with different trophic status had differences. The percentage of adsorbed RS-P was 13.9% in Gonghu Bay of Taihu Lake and had a significant relationship with *Q*_m_ (*p* < 0.05). In East Dongting Lake and Huangda Lake, Ca–P and Fe–P had significant relationship with *Q*_m_ (*p* < 0.05). The percentage of adsorbed Ca–P and Fe–P in East Dongting Lake and Huangda Lake reached 29.9% and 38.2%, respectively.

**Table 5.** The distribution of P fraction and after sorption in control sediments. Note: initial P concentration for sorption was 20 mg l^−1^, and the temperature was 25°C; the contents of P fraction in control sediments and sediments after P sorption, and percentage of adsorbed P fraction are expressed as means ± standard deviation. T, DT and HD stand for the sediments from Gonghu Bay, East Dongting Lake and Huangda Lake, respectively.

sample	P fraction	control sediments (mg kg^−1^)	sediments after P sorption (mg kg^−1^)	percentage of adsorbed P fraction (%)	relationship between *Q*_m_ and P fraction
T	S/L-P	3.8 ± 0.5	75.3 ± 8.2	49.5 ± 7.2	0.783*
	Al–P	89.3 ± 7.2	123.2 ± 10.2	23.5 ± 3.2	0.872*
	Fe–P	198.4 ± 23.2	218.4 ± 30.2	13.9 ± 2.3	0.763
	Ca–P	147.3 ± 19.2	146.2 ± 18.6	−0.9 ± 0.1	0.532
	RS-P	143.2 ± 15.2	163.2 ± 20.1	13.9 ± 2.4	0.623*
DT	S/L-P	12.5 ± 1.1	87.3 ± 7.2	31.9 ± 6.3	0.782*
	Al–P	112.2 ± 20.3	198.3 ± 21.4	36.7 ± 8.2	0.753*
	Fe–P	178.3 ± 26.8	177.2 ± 29.3	−0.5 ± 0.2	0.732
	Ca–P	167.2 ± 19.2	237.2 ± 19.3	29.9 ± 5.3	0.852*
	RS-P	111.8 ± 21.5	116.3 ± 10.6	1.9 ± 0.4	0.523
HD	S/L-P	1.2 ± 0.3	67.3 ± 12.3	44.4 ± 8.9	0.842*
	Al–P	67.3 ± 17.3	97.2 ± 17.6	20.1 ± 5.2	0.729*
	Fe–P	86.3 ± 10.2	143.2 ± 10.2	38.2 ± 3.6	0.793*
	Ca–P	178.4 ± 22.3	175.3 ± 30.5	−2.1 ± 0.5	0.672
	RS-P	162.3 ± 19.7	161.4 ± 27.8	−0.6 ± 0.3	0.562

*p < 0.05.

### 3.5. EPC_0_ of the sediments and soluble reactive P in pore water with different trophic status

Depending on the dynamic equilibrium between solution and solid phase, P can be adsorbed or desorbed from sediments. The trophic status might be one of the main factors of P release in sediment. In this study, the sediment EPC_0_ value at different temperatures and the soluble reactive P concentration in pore water (*C*_pwp_) in four seasons were compared with each other to determine the role of sediments in P release. Table 6 shows the values of ECP_0_ and *C*_pwp_ in sediments from lakes with different trophic status, and the soluble reactive P concentration in overlying water (*C*_owp_) was also determined in four seasons. The ECP_0_ values in Gonghu Bay of Taihu Lake were lower than *C*_pwp_ at 5, 15 and 25°C, whereas higher than *C*_pwp_ at 35°C. As a lightly eutrophic lake, East Dongting Lake had a relatively high *C*_pwp_ (0.081–0.093 mg l^−1^) in four seasons, which caused EPC_0_ to reach values lower than *C*_pwp_ except for 5°C. Huangda Lake had the lowest *C*_pwp_ of the three lakes, and the values varied from 0.039 to 0.053 mg l^−1^. The EPC_0_ values at 5 and 25°C were higher than *C*_pwp_ and at 15 and 35°C had an opposite tendency.

**Table 6.** Zero equilibrium P concentration (EPC_0_) and soluble reactive P concentration in pore water (*C*_pwp_) and overlying water (*C*_owp_) at different temperatures. Note: *C*_pwp_ and *C*_owp_ values were determined in winter, spring, autumn and summer and compared with EPC_0_ values at 5, 15, 25 and 35°C, respectively. T, DT and HD stand for the sediments from Gonghu Bay, East Dongting Lake and Huangda Lake, respectively.

temperature/months	sample	EPC_0_	*C*_pwp_	*C*_owp_
5°C (winter)	T	0.139 ± 0.013	0.098 ± 0.009	0.032 ± 0.004
	DT	0.107 ± 0.014	0.087 ± 0.007	0.021 ± 0.003
	HD	0.055 ± 0.011	0.051 ± 0.002	0.012 ± 0.001
15°C (spring)	T	0.103 ± 0.018	0.068 ± 0.004	0.021 ± 0.012
	DT	0.081 ± 0.011	0.081 ± 0.006	0.039 ± 0.009
	HD	0.039 ± 0.009	0.053 ± 0.002	0.019 ± 0.002
25°C (autumn)	T	0.104 ± 0.018	0.059 ± 0.009	0.021 ± 0.011
	DT	0.087 ± 0.010	0.093 ± 0.006	0.028 ± 0.009
	HD	0.041 ± 0.013	0.039 ± 0.002	0.018 ± 0.003
35°C (summer)	T	0.053 ± 0.009	0.090 ± 0.004	0.053 ± 0.027
	DT	0.025 ± 0.006	0.081 ± 0.004	0.023 ± 0.010
	HD	0.013 ± 0.003	0.051 ± 0.003	0.019 ± 0.002

## 4. Discussion

In this study, kinetic models, modified classic sorption models and thermodynamic models were used to analyse the mechanism of P sorption of the sediments from lakes with different trophic status. In the first few minutes, the active sorption sites were occupied rapidly, and the *Q_t_* increased within 60 min. In the next few hours, the active sorption sites reduced and the P sorption rate decreased. The three sediments had similar kinetic trends, but differences were observed in sorption capacity. The P sorption kinetic results indicate that in autumn, the sediments had higher sorption capacities than in other seasons, and that the sediments in winter had the lowest sorption capacities. The temperature is one of the main factors which could influence the sediment sorption capacities [29,31], and this factor might cause the high P sorption capacities of the sediments in autumn. In addition, the decomposition and sedimentation of phytoplankton or algae occur after summer (in autumn). The OM content in sediments rises, and the P sorption capacity also increases. The OM content is also one of the main factors that can influence the sorption capacities of the sediments [32], and the sediments from Huangda Lake and East Dongting Lake have the higher OM contents. Furthermore, Fe, Al and Ca oxide favoured the sorption capacities of the sediments [33,34]. The sediments from East Dongting Lake had the highest Ca oxide content among the three sediments, and P content in sediments from Huangda Lake was relatively low. Therefore, the sediments from East Dongting Lake and Huangda Lake had higher sorption capacities than those from Gonghu Bay of Taihu Lake. This might be one of the reasons for the low trophic status of East Dongting Lake and Huangda Lake.

The isotherm fitting results indicated that the modified Langmuir model can describe the P sorption isotherm of these sediments, and the Langmuir sorption isotherm also provides a good estimate of theoretical sorption maxima (*Q*_m_), which can reflect the sorption capacities of these three sediments from lakes with different trophic status. Results of thermodynamic analysis indicate the P sorption of the three sediments was spontaneous because of the negative value of Δ*G*^0^ [29], and the decrease in Δ*G*^0^ with the increase in temperature indicates a higher sorption impetus at higher temperature [35,36]. In addition, the sediment from Huangda Lake has the highest sorption capacity because it has the highest Δ*G*^0^ values compared to the other two sediments. The Δ*G*^0^ values also increased as the initial P concentration increased, and the desorption might occur more easily than sorption at the same temperature [37].

Our previous study indicated that the EPC_0_ values can vary with environmental conditions [1]. The sediments from regions with different trophic status at different season may have different EPC_0_ values. As the moderately eutrophic lake, Gonghu Bay of Taihu Lake, has the highest EPC_0_ among the other two lakes, the sediments from Gonghu Bay of Taihu Lake might have more risk of P release than other two lakes. In this study, the EPC_0_ values at 5, 15 and 25°C were compared with the *C*_pwp_ of sediments in winter, spring and autumn, respectively. At 5, 15 and 25°C, the EPC_0_ values were higher than *C*_pwp_, and theoretically, when EPC_0_ > *C*_pwp_, the sediments may release the P, and the P concentration in overlying water (*C*_owp_) increases. However, although the sediments act as a source of P, the *C*_owp_ values are relatively low in winter, spring and autumn. In Gonghu Bay of Taihu Lake with moderately eutrophic status, large amounts of algae exist in water or sediments, and the algae at approximately 5, 15 and 25°C are in dormant, recruitment or growth periods. The P availability increases in these three periods. Therefore, *C*_pwp_ and *C*_owp_ values at 5, 15 and 25°C were lower than at 35°C, which caused the relationship EPC_0_ > *C*_pwp_. When the temperature increases to 35°C, the algae reach the decline period. Large amounts of dead algae release P, and the P concentration increases in water and sediments, resulting in EPC_0_ < *C*_pwp_. The sediment acts as a pool of P, and the sediment might have the risk of P release in summer. In addition, the great accumulation of S/L-P and AL–P fraction in sediments from Gonghu Bay of Taihu Lake after P sorption (table 5) indicated that S/L-P and Al–P were the P fractions, which can participate in the P sorption easily under the moderately eutrophic conditions. Furthermore, in Gonghu Bay of Taihu Lake, the accumulation of RS-P in sediments reflected that RS-P can influence the sorption capacity of sediments in moderately eutrophic lake.

East Dongting Lake is a lightly eutrophic lake in the East Plain of China, and its TSI has increased gradually in recent years. The risk assessment results indicated that the P release in sediments is different from Gonghu Bay of Taihu Lake. At 5 and 15°C, the sediments act as a source of P, whereas at 25 and 35°C, they act as a pool of P. The P concentration in pore and overlying water changes little throughout the year, and the EPC_0_ values vary as the temperature increased. Most of the sediment samples from East Dongting Lake are located near the estuary of the river, where different types of sediments from other sources came together. The sediments have different roles because of the different physicochemical characteristics such as surface area or Ca oxide. Ca–P contributed to the sorption greatly in sediments from East Dongting Lake compared with other two lakes. Most of the sediments from East Dongting Lake are arenaceous, and the Ca oxide content is also higher than other two lakes (table 1). In addition, the low pH of the sediments from East Dongting Lake caused the transformation of adsorbed P into Ca–P [38]. Previous studies suggested that Fe–P and Al–P can be regarded as labile P pools [39].

Huangda Lake is a clean lake with moderately trophic condition and has been relatively stable in recent years. Obviously, the sediments in Huangda Lake act as a pool of P at 15 and 35°C. The change of P concentration in pore water and overlying water in Huangda Lake was similar to that in Gonghu Bay of Taihu Lake. The P concentration in pore water at 25°C was lower than at the other temperatures. In the clean lake with moderately trophic status, the sediments with low TN or TP contents have fewer pollutants (table 1), which might have caused the ECP_0_ values in sediments from Huangda Lake to be lower than in the sediments from the eutrophic lake. At 5°C, EPC_0_ > *C*_pwp_, which illustrates that the sediments in Huangda Lake acted as a P source at low-temperature conditions. Furthermore, the accumulation of Al–P and Fe–P in sediments from Huangda Lake was greater than in sediments from the other two lakes at 25°C, which indicated that large amounts of newly added P remain in the sediments from Huangda Lake. Therefore, the sediments in Huangda Lake might be a pool of P. The result of risk assessment of sediment P release (table 6) indicates that EPC_0_ was larger than *C*_pwp_ in Huangda Lake, and this can also indicate that the sediment acts as a pool of P.

## 5. Conclusion

The sediments from lakes with different trophic status have different sorption characteristics, with the sediment from a moderately trophic lake having a higher sorption capacity compared with the sediments from eutrophic lakes. The environmental temperature can influence the P sorption in sediment, and the sediments in lakes play different roles in four seasons with different temperatures. The P fractions also have different relationships with sorption capacity in sediment with different trophic statuses. In the sorption process, S/L-P and Al–P were the P fractions which had a significant relationship with sorption capacity in sediments from all three lakes. Ca–P and Fe–P had a significant relationship with sorption capacity of sediments from lightly eutrophic and moderately trophic lakes. In addition, the sediments from moderately trophic lakes in summer have the highest P sorption capacity. The sediments from the moderately eutrophic lake acted as a source in summer, and acted as a pool in other seasons. In the lightly eutrophic and moderately trophic lakes, the sediment had the risk of P release in spring and winter while acting as a pool in autumn and summer. With three regions of different trophic status as examples, this study revealed the P sorption characteristics of sediment at different seasons to provide a theoretical foundation for the control of P release in sediments from different trophic statuses.

## Data accessibility

Data are available on Dryad: http://dx.doi.org/10.5061/dryad.kt14vq3 [40].
